# Engaging Patient and Caregiver Partners in Codeveloping a Patient Educational Video for Improving Clostridioides difficile Infection Education: Participatory Co-Design Study

**DOI:** 10.2196/81643

**Published:** 2026-03-04

**Authors:** Ritika Kamlesh Patel, Rajvir Teja, Kristy Hermann, Rose Franz, Karen Wong, Dina Kao

**Affiliations:** 1 Division of Gastroenterology Faculty of Medicine and Dentistry University of Alberta Edmonton, AB Canada; 2 Faculty of Nursing University of Alberta Edmonton, AB Canada

**Keywords:** Clostridioides difficile infection, patient educational resources, digital health, fecal microbiota transplant, codevelopment, informational video

## Abstract

**Background:**

Patients with recurrent *Clostridioides difficile* infection (rCDI) and their caregivers often face considerable uncertainty regarding medical management, including the use of fecal microbiota transplantation (FMT), largely due to the scarcity of accessible and credible educational resources. Codeveloping educational materials with patients and caregivers offers a structured way to address these gaps and ensure that resources reflect the informational, psychological, and emotional needs of patients.

**Objective:**

The study team sought to cocreate an educational resource through an iterative process including patient and caregiver partners with lived experience of rCDI to improve *C difficile* infection education.

**Methods:**

This study examined the cocreation process of a patient-centered educational resource between the study team and patient or caregiver participants through a series of focus group (FG) sessions. Five participants took part in 3 serial FG sessions (3-5 participants each) over 13 months. Each FG session was audio recorded, transcribed, and analyzed using the NVivo (version 14) quantitative analysis software. A semantic thematic analysis framework was applied to interpret the results. Key areas of concern and preferred formats were identified following the first FG session. The first version of the educational resource was developed by the study team to address areas of concern and was iteratively refined following feedback from subsequent FG sessions with the study participants.

**Results:**

Participants expressed concerns about the lack of credible information on treatment options and their associated risks, especially with regard to FMT. They noted inadequate coverage of *C difficile* infection recurrence and its physical, psychological, and emotional impacts. Participants expressed a preference for educational resources in video format. On the basis of further feedback, refinements were made to improve pacing, consistency of animation, and narration, incorporating emotional and mental health considerations. The codeveloped video was well received and valued for its clear language, messaging, step-by-step guidance, and overall accessibility and clarity.

**Conclusions:**

Our study demonstrated the feasibility and utility of patient and caregiver involvement in cocreating an educational resource on the management of rCDI, with FMT being a treatment option. Despite efforts to address knowledge gaps and preferences expressed for a video format, uncertainties remain regarding the most effective educational resource format. The integration of patient, caregiver, and study team perspectives contributed to a codeveloped video that addresses unmet needs and is patient centered. However, diverse patient experiences remain underrepresented. Future research should consider including more diverse participants as well as evaluating the effectiveness of knowledge improvement through various educational resource formats and patient health care experiences and levels of satisfaction.

## Introduction

Commonly triggered by the use of antibiotics, *Clostridioides difficile* infection (CDI) is a gram-positive bacterial infection that occurs due to intestinal dysbiosis. The first-line therapy for CDI is vancomycin, which further exacerbates dysbiosis. Consequently, the risk of CDI recurrence continues to rise, from 20% to 30% after the first episode up to >60% after the third episode [1,2].

Fecal microbiota transplantation (FMT), a procedure that transfers stool from a rigorously screened donor into the intestine of a recipient, is a highly effective, guideline-recommended therapy for managing recurrent CDI (rCDI). The efficacy is at least 80% after a single treatment [3]. However, awareness and understanding of FMT remain limited among patients and health care providers, particularly regarding what the procedure entails, what the potential risks are, when FMT is indicated, or how to access it.

Although the global burden of CDI has consistently increased over the last 20 years [4], there is still a lack of credible and evidence-based resources available to the public [5]. Many patients seek information from unreliable sources [6] or delay seeking medical attention [7]. Studies exploring patient experience with rCDI have identified confusion and uncertainty about symptoms as predominant concerns among patients and their families [8,9]. Additionally, surveyed patients emphasized the importance of understanding their condition and expressed a desire for greater access to educational resources. When asked about their preferred methods of learning, patients indicated a preference for verbal and written education supplemented with online resources [8]. The illness experience is impacted by various factors, including prior experiences and the quality of care received. Notably, patients cited their lack of knowledge and understanding as negative contributors to their overall experience with rCDI [8].

Patients and health care professionals often have differing priorities when it comes to research and resource development. While health care professionals prioritize communication and effectiveness, patients tend to focus on psychosocial consequences and human behavior [10]. Patient partnerships are grounded in experiential knowledge, defined as “the knowledge embedded in the patient’s experience of managing and living with an illness” [11]. Patient partners view themselves as equal members of the health care team and become more engaged in their own health due to their participation; they are further motivated by the opportunity to improve care quality for others. As such, patient partners play a crucial role in the resource development process by integrating patient perspectives to ensure that resources are tailored to the populations they are designed to serve [12]. Ultimately, the final product will be shaped by both the scientific and experiential knowledge of health care professionals and the lived experiences of patients [13].

Given the paucity of credible, patient-centered educational resources for the management of rCDI, this paper describes the iterative process of codeveloping an educational video by patient and caregiver partners with health care providers and how the final product was perceived and disseminated.

## Methods

### Design

The study team from the FMT program in Edmonton, Alberta, aimed to cocreate a patient-centered educational resource to guide patients and caregivers through rCDI treatment. A qualitative approach was taken, using serial focus group (FG) sessions to collect data to inform the format and content of the educational resource. Three 60-minute FG sessions with patient partners and caregivers were conducted, facilitated by the Alberta Health Services (AHS) Health Systems Knowledge and Evaluation (HSKE) team. The sessions followed a semistructured format framed by an interview guide in addition to open-ended questions. A separate interview guide was designed for each FG. The final product was the result of an iterative process of cocreating an educational video with important feedback from patient partners and caregivers on their experiences with rCDI and FMT.

### Ethical Considerations

Ethics approval was obtained from the Health Research Ethics Board of the University of Alberta (Pro00130489), where the study was conducted. Informed consent was obtained after explaining the study protocol and the voluntary nature of involvement to all participants. Each participant signed a written consent form that explicitly covered both study participation and the audio recording of FGs. Financial compensation was provided to participants in the form of gift cards. All data were deidentified to protect the privacy of participants and maintain confidentiality.

### Participant Recruitment

Participants were recruited through convenience sampling, as they were former patients of the single FMT clinic in Edmonton. Eligibility criteria included having previously received FMT or having cared for an FMT recipient. The FMT clinic nurse navigator contacted eligible individuals to provide an overview of the study and assess their interest and availability to participate in 3 FGs. Upon receiving written consent for participation and audio recording, virtual meeting invitations were distributed. While all participants contributed meaningfully to the study, attendance varied across the FGs.

### Data Collection and Analysis

Zoom (Zoom Video Communications) was used for FG 1, which took place on October 15, 2023. Microsoft Teams was used for FG 2 on August 15, 2024, and FG 3 on November 5, 2024. Each FG lasted approximately 60 minutes. Sessions were transcribed using the auto-transcription features on both Zoom and Microsoft Teams. The first session served as an opportunity to explore patient experiences with rCDI, their emotional responses throughout the illness, access to educational resources, preferred formats for the resources, and the most effective methods of dissemination. The purpose of the initial FG was to establish a baseline understanding of the current landscape of educational resources and patients’ perspectives of them. The second and third FGs were used to gather insights and opinions from participants to involve them in the cocreation of the educational resource.

In response to this feedback, the study team developed the initial draft of the educational video and shared it with participants 2 weeks before FG 2.

During the second FG, participants were asked to provide feedback on ways to improve the educational resource. Their responses, combined with input from the AHS Clinical Knowledge and Content Management team, were compiled and used to refine the educational resource further. A similar process was followed before FG 3, where the revised product was sent to the participants who had contributed to FG 2. They were asked to review the updated product and provide feedback on whether the modifications adequately addressed their concerns from FG 1. The study team revised the educational resource based on the feedback and provided a revised version to the participants 2 weeks before FG 3.

To prepare the data for analysis, all FG transcripts and interviews were reviewed and cleaned for accuracy by the HSKE team. Identifying patient information was replaced with study identifiers to maintain participant confidentiality. Thematic analysis was used to examine the qualitative data extracted from the FG transcripts. The NVivo software (version 14; Lumivero) was used to aid in coding the data by the HSKE team using a semantic approach, ensuring that participant perspectives were accurately captured. A semantic approach to thematic analysis was considered favorable for this study, as it involves coding data based on explicit content, resulting in a descriptive rather than interpretative analysis [14]. This approach was particularly useful in this context, as it facilitated the categorization of patient suggestions into various thematic groups, allowing for their seamless integration into the final version of the educational resource.

## Results

### Participants

Of 20 patients and caregivers approached, 7 (35%) participants were recruited (n=6, 86% patients and n=1, 14% caregivers). Ultimately, 2 (29%) participants withdrew before initiating the study due to other competing responsibilities, time constraints, or scheduling conflicts at the time of the FGs. Of the 5 participants from FG 1, a total of 3 continued with participation in the subsequent FGs. Attrition following the initial FG occurred because participants were unable to commit to additional time. Baseline participant characteristics are summarized in Table 1.

**Table 1:** Summary of participant demographics by focus group.

Characteristic			Focus group 1 (n=5)					Focus groups 2 and 3 (n=3)—patients
			Patients		Caregivers			
Male, n (%)			2 (40)		0 (0)		2 (67)	
Age (years), mean (SD)			49 (13.5)		53 (N/A^a^)		43 (8.7)	
**Educational level, n (%)**								
	University	2 (40)		1 (20)		2 (67)		
	Nonuniversity postsecondary education	1 (20)		0 (0)		0 (0)		
	High school	1 (20)		0 (0)		1 (33)		
**Ethnicity, n (%)**								
	South Asian	1 (20)		0 (0)		1 (33)		
	Southeast Asian	1 (20)		1 (20)		1 (33)		
	White	2 (40)		0 (0)		1 (33)		
**Comorbidities, n (%)**								
	Multiple comorbidities^b^	1 (20)		0 (0)		1 (33)		
	Cognitive impairment	0 (0)		0 (0)		0 (0)		
**Living situation, n (%)**								
	Independent	4 (80)		1 (20)		3 (100)		

^a^N/A: not applicable (as only 1 participant was present).

^b^Includes type 2 diabetes, dyslipidemia, kidney transplant for end-stage renal disease, hyperparathyroidism, hypophosphatemia, and coronary artery disease.

### FG Discussions

Three FG sessions were conducted between October 2023 and November 2024. The findings highlighted key areas of concern for patients with rCDI, including previously unknown information, content that patients found valuable, and gaps in coverage and representation in existing resources. A summary of FG discussions can be found in Table S1 in Multimedia Appendix 1.

In FG 1, participants reported feeling frustrated and confused while searching online resources about FMT and rCDI. While some information was perceived as credible and trustworthy, other content was considered too generic, inaccurate, or outdated, leading to uncertainty about its reliability (Table S1 in Multimedia Appendix 1):

I found while there were very trustworthy sources online like the World Health Organization and the US Centers for Disease Control and some other Canadian health jurisdictions as well, in the United States, there were an equal number of sources that may not be considered as trustworthy. So, I found that doing research online was a little bit nerve wracking because you didn’t know what treatment options were accurate, when the documents were last updated, so maybe there was an outdated treatment method as well, so I found that to be more concerning looking stuff up online than actually informative.

Participant 2

A common issue identified was the lack of awareness of FMT among physicians and associated delays in its implementation as a treatment option. Participants were alarmed that primary care providers appeared to be unfamiliar with FMT as a treatment option, despite thinking rCDI was a prevalent illness (Table S1 in Multimedia Appendix 1):

My doctor didn’t have that information either, and I’m the one that brought it up. I’m the one that researched Dr. X and then when I took it to her, she says, “yeah, OK.” So, the lack of information out there in regards to the fecal transplant is, I find very alarming.

Participant 3

Participants identified several important topics for educational resources for patients with rCDI, including causes of rCDI, signs and symptoms, available treatment options, potential risks, and when to seek treatment (Table S1 in Multimedia Appendix 1):

I think the signs and the symptoms of it, if people know what to look for because I didn’t know, I thought I’d eaten something bad, and it wasn’t that at all.

Participant 1

If it was known that [there] is a high possibility of it reoccurring. That’s good information to have and, I know I had to be an advocate for my own health because, I knew it, I wasn’t better.

Participant 3

Furthermore, participants emphasized the importance of raising awareness about the significant likelihood of the recurrence of CDI and its impact on mental health (Table S1 in Multimedia Appendix 1):

For me it was not only challenging physically to deal with so much of it, but mentally as well. And there were times, I was thinking, oh my goodness. When is this going to end?... I thought, every now and then, you know what, I need to give up on this, this is just too much. So, for me, I feel that having information about not giving up, surrounding yourself with people you love, and who love you back, is important as well.

Participant 2

Participants felt that a video format would offer the most comprehensive approach, as it allows for easy accessibility and can effectively capture and maintain viewer attention when hosted on trusted platforms such as the AHS website. At the same time, they expressed a desire for mixed-format patient educational resources in the future (Table S1 in Multimedia Appendix 1):

For digital resources, I feel that since a lot of people are using social media these days, like TikTok, Instagram, Facebook.... I think it was [Participant 4] who mentioned having little ads on TV, I feel that having little ads on social media as well that refer the user to go to the Alberta Health Services website or My Health. That can help as well.

Participant 2

I love those small pamphlets that you take from a plastic thing, and then you open it...information’s there on FMT and then the marketing would be on the front page, something like “been pooping lately?” And then at the bottom something like “try FMT” and then as you open the page “what is FMT?” with some short information on it. Because I got lots of that before [my] kidney transplant. And boy, did I learn a lot from it.

Participant 5

On the basis of the feedback from FG 1, a video about CDI and FMT was considered the preferred format. Patient partners emphasized that representation was a key priority, noting a significant lack of diversity within existing educational resources. As a result, the development of new materials needed to intentionally incorporate principles of diversity, equity, and inclusion, ensuring that content reflected a broad spectrum of socioeconomic backgrounds, genders, and other underrepresented subgroups. To incorporate this feedback, the resource was drafted using inclusive language and written at an eighth-grade reading level to ensure accessibility for a broad audience. Additionally, both male and female narrators were included in the video to enhance representation and reflect greater diversity.

Before FG 2, the study team developed a script and storyboard for an animation-style video in a question-and-answer format (Table S2 in Multimedia Appendix 1). Key topics included a holistic perspective on managing CDI, benefits and limitations of FMT, patient expectations associated with FMT, and prevention of future CDI. This draft video was shared with participants 2 weeks before FG session 2.

Participants in FG 2 expressed that the video was clear and concise and effectively addressed questions that patients might have. They also found that the video was easy to follow, engaging, user-friendly, and free from complex medical jargon (Table S3 in Multimedia Appendix 1):

I found it to be very clear and to the point, effectively conveying the most important information. So, it basically answered most of the questions I would have if I were still a patient and I appreciated that it explained the screening process for donors since this can provide additional comfort to patients.

Participant 2

It’s not jargon. It’s not medical jargon. It’s not too simple. It’s not too complex.

Participant 7

The participants expressed a desire for consistency in both the visuals and audio. They noted that the male and female speakers had differing audio qualities and volumes, which made the overall balance feel off. Furthermore, all participants pointed out that there were moments in the video in which the audio seemed to move faster than the images and the slide transitions were too rapid (Table S3 in Multimedia Appendix 1):

Yeah, I noted that same thing as well, where the female narrator was louder than the male narrator. I was constantly having to fiddle with the volume controls on my laptop just to compensate for that.

Participant 2

Participants felt that the amount of information presented in the video about rCDI and FMT was appropriate for the length of the video. However, they thought that the video could be longer (Table S3 in Multimedia Appendix 1):

[F]or information with this much value, I don’t think it would be much of an issue to extend the video by maybe another minute or so.

Participant 2

Participants did recommend adding more information and indicating specific timestamps (eg, 3:35) in the portion of the video that talked about recurrence of CDI to improve clarity (Table S3 in Multimedia Appendix 1):

For myself, I found the information to be quite clear. However, there was one specific question. “How do you know if the FMT was successful?” and the answer was if the diarrhea does not come back, then you can consider yourself cured.... But I found that to be quite vague, since there is no window of recurrence specified. So, if the diarrhea does not come back in three days are they cured? or three weeks? etcetera.

Participant 2

Participants also requested more details about the potential risks of FMT and sought clarification on the process used to handle the fecal matter after it is collected from the donor. The script was consequently updated to reflect these preferences (Tables S3 and S4 in Multimedia Appendix 1):

I’m not sure if I saw this in the video, but what are the risks of the FMT...? I don’t think that was covered.

Participant 2

[W]e could potentially think about adding a little bit...about...how it goes from donor to the capsule or to, whatever form you’re using. So, it might be valuable to say that this is fecal filtrates, or that it’s processed in a certain way...or whatever you want to say. We don’t have to get scientific, but it would be nice so that they know there’s a process to refining it so that it’s not just stool to you, right?

Participant 7

Participants were shown examples of health education on the website of MyHealth Alberta and asked for feedback on the most and least helpful formats. They preferred question-and-answer and video formats but suggested offering information in multiple formats to accommodate various audiences and situations, such as providing video transcripts for those unable to watch videos in public (Table S3 in Multimedia Appendix 1):

I really like the Q&A style that’s then augmented by having the video, which is further augmented by having that transcript, if that’s how you prefer to learn, or hear it, or view it, or whatever.

Participant 7

In FG 3, participants felt that the audio and visual issues from the second FG were resolved and appreciated the additional information provided in the video. However, they agreed that the term “fecal slurry” was unpleasant. The visuals were deemed suitable for CDI-affected audiences, except for the image of the glass of fecal slurry. The animations accompanying this portion of the video were changed before submitting the video to the MyHealth Alberta website for publication (Table S5 in Multimedia Appendix 1):

Yeah. I didn’t expect anything more than that. I felt it covered it very well in terms of specifically the composition of the FMT capsules, which was interesting to see.

Participant 2

And then that “fecal slurry,” that was an interesting picture. Some people might find that too much information.

Participant 7

**Figure 1:**
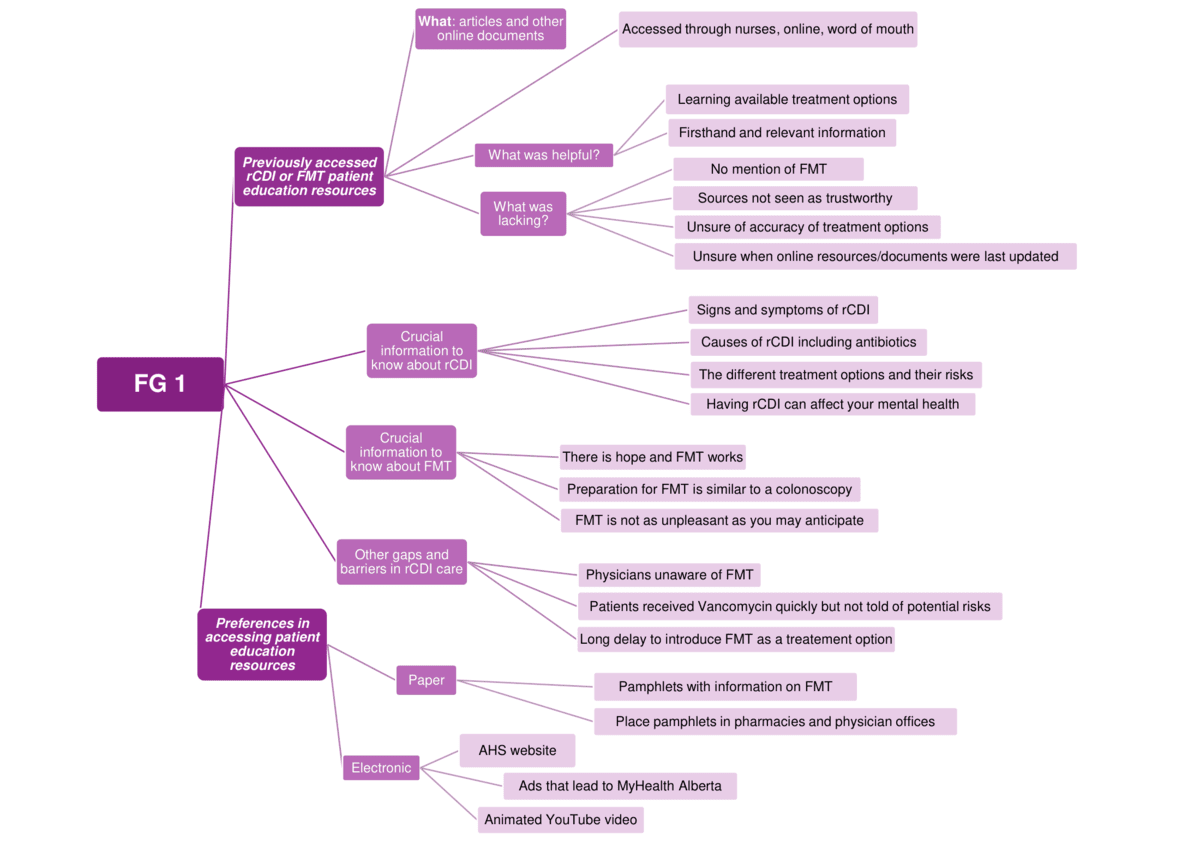
Thematic map of focus group session 1 (FG 1). AHS: Alberta Health Services; FMT: fecal microbiota transplantation; rCDI: recurrent Clostridioides difficile infection.

**Figure 2:**
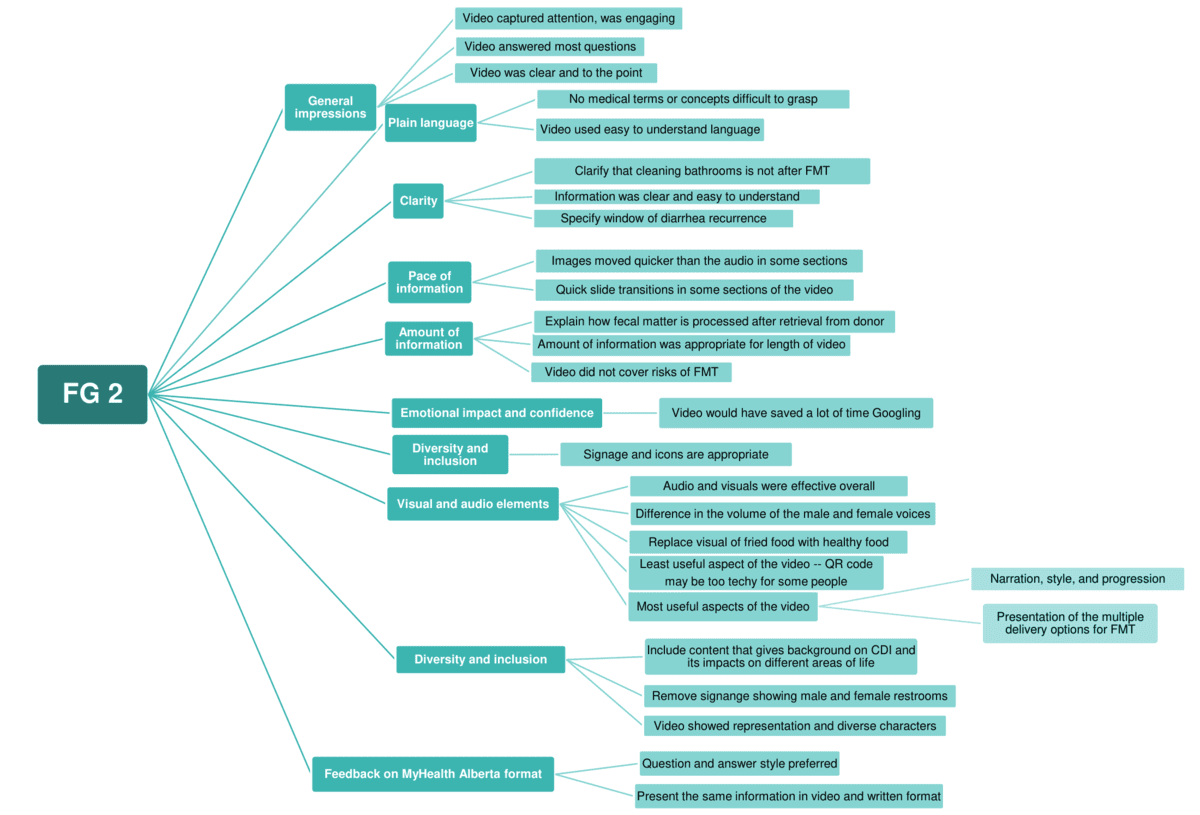
Thematic map of focus group session 2 (FG 2). CDI: Clostridioides difficile infection; FMT: fecal microbiota transplantation.

**Figure 3:**
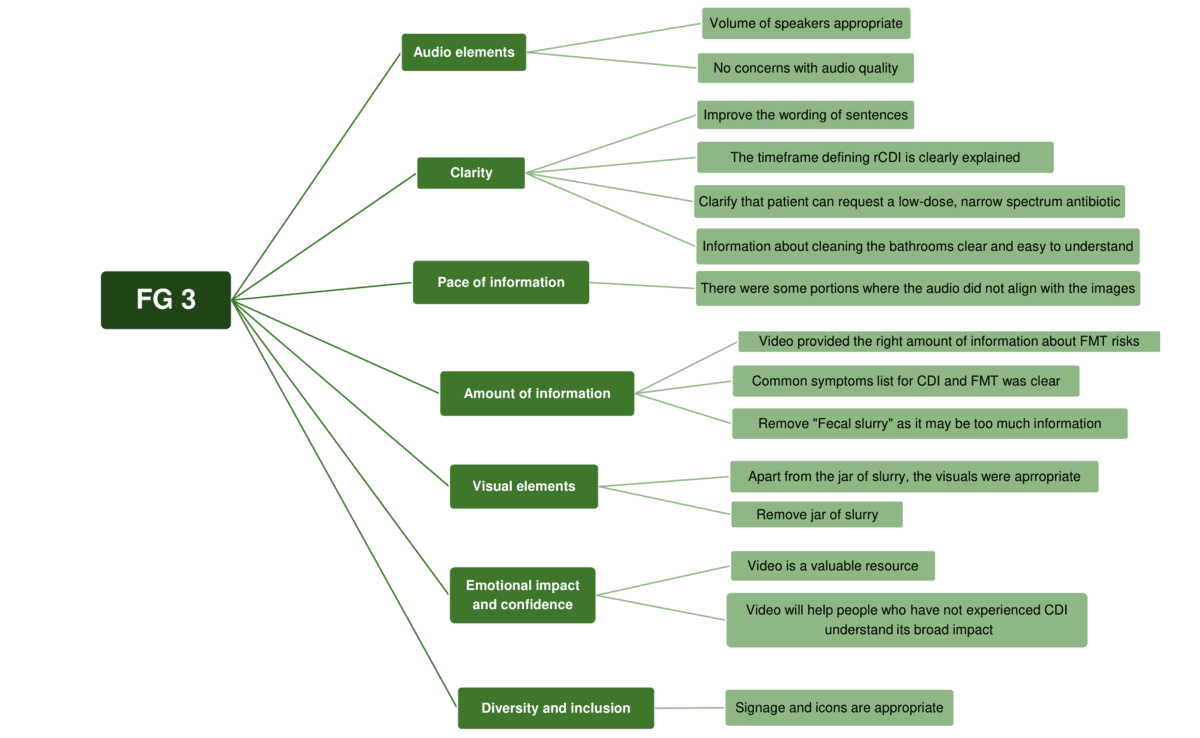
Thematic map of focus group session 3 (FG 3). CDI: Clostridioides difficile infection; FMT: fecal microbiota transplantation; rCDI: recurrent Clostridioides difficile infection.

Thematic analysis of the FG sessions reflected informational needs, emotional and practical challenges associated with rCDI, and preferences regarding the delivery of educational content (Figures 1-3). Participants emphasized the importance of clarity, reassurance, and transparency surrounding treatment risks and benefits within the video content. In addition, preferences related to video pacing, diction style, and audiovisual elements were identified. The finalized video was published on MyHealth Alberta in February 2025.

## Discussion

### Principal Findings

Patient and caregiver partners in our study identified the inadequacy of existing educational resources in addressing their concerns throughout their rCDI journey. Using an iterative cocreation process with our partners, we developed an animated patient-centered video focused on rCDI and FMT, the most effective therapeutic option. This video was regarded by our partners as medically informative, relevant, reassuring, and inclusive while authentically capturing patient perspectives, demonstrating the value and feasibility of patient-centered, cocreated educational resource development.

### Current Landscape

Low health literacy is strongly associated with worse health outcomes and higher health care costs [15]. Patient educational materials are *actionable* when “consumers of diverse backgrounds and varying levels of health literacy can identify what they can do based on the information presented” [16]. Furthermore, pictograms have been shown to improve the acquisition and comprehension of complex medical information [17]. Multiple surveys of patients with CDI and health care providers have found that education provision is inconsistent [18,19]. While educational resources on rCDI exist, they often fail to reflect patient experiences, contributing to frustration and a lack of understanding [16]. A systematic appraisal of 19 educational materials for patients with CDI using the validated Patient Education Materials Assessment Tool found that the understandability averaged at 73.4 (range 62.7-82.9), whereas the overall actionability score was 50.5 (range 13-73.3), and none used visual aids [20]. While the American Gastroenterological Association [21] released an educational video in 2024 specifically on FMT, it has limited patient engagement and does not address many concerns raised by our study participants, including risks associated with FMT, what to expect after FMT, or how to prevent future recurrence.

Perspectives shared by participants reflect a broader pattern of underuse of FMT despite its endorsement in clinical practice guidelines. A survey including 31 hospital-based FMT centers across 17 European countries reported a total of 1874 FMT procedures in 2019, of which 1077 (57%) were performed for CDI. In contrast, an estimated 12,400 (range 6100-28,500) annual cases of multiply rCDI with an indication for FMT occur in Europe, suggesting that current FMT activity reaches only approximately 10% of eligible patients [22]. Several factors likely contribute to this discrepancy between guideline recommendations and real-world practice. Knowledge-related barriers [23,24] among health care professionals may include unfamiliarity with FMT as a treatment and its efficacy or uncertainty regarding the timing of referral or which specialists to refer to. In addition, a 2024 survey of infectious disease physicians and other health care professionals in the United States found that although 87% of respondents reported having previously recommended FMT for rCDI, only 48% [25] indicated having access to FMT using donor stool. Recent approval of Rebyota, a donor stool–based microbial therapy, by the Food and Drug Administration and Health Canada may potentially improve access to treatment.

### Target Population

Participants in our study may appear younger and healthier than the traditional CDI population, once linked to hospitalized, older, frail, or comorbid adults. However, approximately 30% of patients with rCDI evaluated in the Edmonton FMT program were aged <50 years without significant comorbidities (D Kao, unpublished data, December 2025). Similarly, emerging evidence from the last 20 years has highlighted a drastic shift in CDI epidemiology worldwide, with more frequent occurrence causing severe disease in populations that were previously thought to be at low risk [26]. Additionally, those with community-acquired CDI tend to be younger [27].

Despite this CDI epidemiological shift, advanced age and frailty continue to be risk factors for CDI, and it is important to consider how to best structure and disseminate educational resources within these populations. As participants in our study were relatively young (mean age of 54, SD 9.3 years), this may have influenced the preference for digital formats, perspectives on self-advocacy, and familiarity with online search engines. As a result, findings from our study may have limited applicability to older adults, particularly those with comorbidities, cognitive impairment, or limited digital literacy. A study exploring digital health literacy among older adults found that respondents scored relatively low in the domains of technology familiarity, technology confidence, and technology incentive [28]*.* While 61% of the respondents in that study experienced no stress while using a computer, 9% of participants experienced high or very high stress levels. At the same time, there is evidence suggesting that technology and mobile device use among older adults is steadily increasing, with rising mobile phone ownership among individuals aged ≥65 years, supporting the feasibility of digital health education in this population [29]. As reliance on digital dissemination alone may be insufficient to reach all populations, particularly older adults and individuals with limited digital literacy, it is important to consider multimodal dissemination strategies to improve patient education.

Although concerns remain regarding older adults’ ability to access digital educational resources, this mode of dissemination offers several advantages that align with findings from our FG sessions. Since the completion of our educational video, it has been uploaded to My Health Alberta, an online health information source built by the government of Alberta and AHS. The link to this video is also embedded in the provincial-based referral pathway for CDI, allowing other health care providers to directly share the resource with patients. In addition, all patients referred to the Edmonton FMT clinic receive a link to this video before their scheduled appointment, as well as support from members of the clinical care team, who assist those experiencing difficulties accessing the video resource.

### Importance of the Cocreation Process

There is increasing recognition that cocreation is essential for developing patient educational resources that are relevant, accessible, and impactful. A study of brain cancer resources that combined content analysis with participant interviews highlighted 4 critical design imperatives: authenticity, visual engagement, concise key messages, and clear information. Participants favored resources that met contemporary visual standards and featured multiple narrators [30], similar to preferences expressed by our study participants. Valuable insights that might otherwise be overlooked can emerge using a cocreation approach. Evidence across the literature shows that cocreation is associated with improved treatment adherence and better health outcomes [31-33]. Moreover, it supports the delivery of patient-centered care by aligning health care services with individual needs and preferences, thereby enhancing overall satisfaction [32,33].

The recurrence and stability of themes across FGs indicate that thematic saturation was reached, supporting the robustness of the insights that informed the cocreation process.

### Comparison of Various Formats

Print pamphlets have long been considered an effective means of disseminating patient education, largely due to their accessibility across socioeconomic groups [34]. However, their high production costs and low rates of patient recall significantly limit their impact [35]. In contrast, clinician-produced videos have demonstrated improvements in decision-making, knowledge acquisition, and anxiety reduction [36,37]. Multimedia formats incorporating slide and sound presentations have been shown to outperform both pamphlets and face-to-face dialogue in facilitating knowledge translation [38]. A cocreated child-narrated video on uveitis showed that self-rated understanding of uveitis, described as “good,” increased from 33% to 51% upon viewing the video. Those who self-rated their understanding as “none” dropped from 19% to 0% [38]. Objective knowledge scores, measured through asking various questions about video content, increased significantly for all 6 questions posed to participants. Notably, a study with patients with psoriasis comparing the effectiveness of pamphlets with that of videos through tests found that post–pamphlet plus video test scores (mean 86.25, SD 17.58) were significantly higher than the postpamphlet test score alone (mean 72.08, SD 26.33; *P*<.001) [24].

There were several reasons for prioritizing a video-based format. First, our participants preferred this format. Second, it can integrate multiple modes of information delivery, including visual text, imagery, and spoken content. Existing literature suggests that incorporating diverse learning modalities may enhance knowledge acquisition [39,40] and retention among patients. Third, there is a limited availability of clinically developed, patient-facing educational videos on CDI that patients can reliably access. Fourth, videos enable participants to pause, rewatch, or visually interpret information if spoken content is unclear, providing greater flexibility in comprehension [41]. While clinician dialogue is valuable, it does not offer unlimited access. Similarly, pamphlets often require distribution through medical visits, limiting accessibility. Outdated pamphlets also require physical replacement, unlike videos. Videos allow patients to review information before attending appointments, potentially enhancing preparedness for discussions with health care providers.

It is important to consider that, when investigating the effectiveness of knowledge transfer, some studies have found that adding an instructional video on bowel preparation before colonoscopy improved preparation quality, while other studies have not [42]. Additionally, digital engagement tends to decline among older adults and individuals with lower educational attainment [43]. These challenges can be mitigated through targeted coaching and clear guidance from health care providers. These strategies can help patients navigate and trust online resources, linking video-based education to improvements in health literacy and more equitable access to information [44-46]. The extent to which any educational tool optimally turns information into lasting understanding and action and subsequent impact remains uncertain [42]. Future studies should incorporate an evaluation process to assess patient comprehension in a systematic manner.

### Limitations

This study has several limitations that should be considered when interpreting the results.

The small sample size of 5 participants limits the generalizability of the findings. A larger sample could have captured a broader range of perspectives, particularly regarding differences in patient experiences across various demographic groups. Given the limited number of participants, those who chose to take part may have had stronger opinions or a greater interest in the development of patient educational resources than the general population of patients and caregivers. This self-selection bias could have affected the topics and content included in our educational resource, which may not be of interest to a more diverse audience.

The use of FGs has inherent limitations, including susceptibility to bias, as dominant participants or the moderator may influence individual or group opinions [47,48]. As a result, distinguishing personal opinions from group consensus can be challenging. However, FGs remain a valuable method in health research, as their interactive nature encourages participants to voice their perspectives, ultimately enhancing data collection [49]. Additionally, FG sessions offer a convenient and effective approach, particularly in an evolving digital health space, to gather meaningful insights into patient experiences, perspectives, and needs [50].

Retention is a well-recognized challenge in qualitative research, which we also encountered, as participants may have had limited capacity to commit to multiple 1-hour sessions due to competing responsibilities, including employment and caregiving obligations. Additional barriers may include technological constraints such as limited access to the internet or devices. In future studies, retention may be enhanced through the provision of multimodal options for FG participation (eg, in-person, virtual, or asynchronous formats), as well as more proactive and individualized communication with participants regarding accommodations that could facilitate continued involvement. Gaining a clearer understanding of individual-level barriers to participation may allow research teams to implement targeted solutions, thereby improving retention and reinforcing participants’ sense of value in the research process [51].

The study sample lacked representation from frail, institutionalized, and cognitively impaired populations. These groups are likely to have distinct educational needs and may rely more heavily on caregivers for health system navigation. To ensure a greater inclusion of diverse perspectives, future studies should implement appropriate recruitment and retention strategies. The literature suggests that multifaceted recruitment approaches, those that provide clear study information, offer incentives in multiple forms (eg, financial, health related, or educational), and are initiated by a trusted member of the health care team, are associated with improved engagement [52]. Furthermore, involving participants in project development has been shown to support retention by fostering a sense of ownership and relevance [51]. While participant involvement was incorporated in this study through a cocreation process, engaging patients more directly in the qualitative design, including feedback mechanisms and FG facilitation, may further reduce attrition [53].

In terms of data collection, a standardized qualitative research checklist, such as COREQ (Consolidated Criteria for Reporting Qualitative Research) or SRQR (Standards for Reporting Qualitative Research), was not used in this study. Incorporation of such frameworks in future research may strengthen methodological transparency and rigor [54,55]. Moreover, the FG guides were not pilot-tested, which may have limited the ability to assess and refine their content validity [56].

### Conclusions

Cocreating educational resources with patient partners is a highly feasible approach that yields patient-centered, well-received tools for managing rCDI. Future work should evaluate the acceptability and effectiveness of additional formats to ensure that diverse patient populations have access to the most engaging and clinically relevant resources.

Given the diversity of patient and caregiver populations and the wide range of individual experiences with CDI, many perspectives remain underrepresented in the current collection of educational materials. To ensure inclusivity and relevance, it is essential to continue collaborating with health care providers, patient partners, and other stakeholders to allow for the continuous evolution of these resources. Further studies should explore the effectiveness of patient educational videos for CDI and FMT in improving patient knowledge, treatment adherence, and overall health care experiences. Additional evaluation studies, both quantitative and qualitative, are necessary to assess the impact of such resources on prevention and treatment outcomes. To determine the effectiveness of different formats of patient education, exploratory qualitative studies could be used to investigate the extent to which codevelopment processes can address existing gaps in CDI-related patient education.

## Appendix

Multimedia Appendix 1 Focus group and video script tables.

## Abbreviations

AHS: Alberta Health Services
CDI: Clostridioides difficile infection
COREQ: Consolidated Criteria for Reporting Qualitative Research
FG: focus group
FMT: fecal microbiota transplantation
HSKE: Health Systems Knowledge and Evaluation
rCDI: recurrent Clostridioides difficile infection
SRQR: Standards for Reporting Qualitative Research
