# Supplementary material for: Engaging Patient and Caregiver Partners in Codeveloping a Patient Educational Video for Improving Clostridioides difficile Infection Education: Participatory Co-Design Study
Source: JMIR Form Res. 2026 Mar 4;10:e81643. doi: 10.2196/81643 (PMC13000376; doi:10.2196/81643)
Supplement: Multimedia Appendix 1 [file formative_v10i1e81643_app1.docx]

**Supplementary Table 1.** Focus Group 1 Responses

| **Question/ Category** | **Sub-questions** | **Summary of responses** | **Sample quotes** |
| --- | --- | --- | --- |
|  |  |  |  |
| 1. Before participating in this study, have you ever accessed any patient education resources about:    1. rCDI    2. FMT? | What were they? | Most participants indicated that they had accessed patient education resources about rCDI/FMT. Resources accessed included articles and other online documents. | *“I found while there were very trustworthy sources online like the World Health Organization and the US Centers for Disease Control and some other Canadian health jurisdictions as well, and in the United States, there were an equal number of sources that may not be considered as trustworthy. So, I found that doing research online was a little bit nerve wracking as well because you didn't know what treatment options were accurate, when the documents were last updated as well, so maybe there was an outdated treatment method as well, so I found that to be more* *concerning looking stuff up online than actually informative.”*   - *Participant 2* |
|  | Where did you find them? | - Participants reported finding online credible sources. - Participants accepted sources as ‘credible’ when they were recognized provincial, national or international organizations e.g., WHO, US CDC, Mayo Clinic, AHS, other Canadian health jurisdictions). - Word of mouth from friends or trusted healthcare professionals. | *“I went online, looked for credible sources. Another one that I went to was the Mayo Clinic.”*   - *Participant 1*   *“I actually got it* [information] *from a friend that had gone through the fecal transplant in 2007.”*   - *Participant 3*     *“My nurse mentioned it.”*   - *Participant 5* |
|  | What did you find helpful? | - Articles that addressed available treatment options for rCDI including FMT. - Information that was firsthand and relevant. | *“A lot of the articles that I read, did mention FMT as a treatment option if taking Vancomycin and the other antibiotic did not resolve the issue,…, and also what other treatment options might be available even if FMT doesn't work.”*   - *Participant 2*   *“What* [information] *was online was very generic and this person* [patient’s friend] *had gone through the whole disease and had gone through the fecal transplant. It was very good information because it was firsthand.”*   - *Participant 3* |
|  | What did you find lacking? | Participants mentioned that the following was lacking about rCDI resources:   - The information was very general. - No mention of FMT as a treatment option. - Not sure about the accuracy of treatment options in the resources. - Not sure when the online resources were last updated. - Lack of information about the risks of treatment options. - Some sources were not seen as trustworthy.   Participants mentioned that the following was lacking about FMT resources:   - Lack of information about FMT from a family physician. | *“*[My information] *was just on C diff. There was no mention of the fecal transplant.”*   - *Participant 1*   *“I found the thing that was least helpful was the fact that when I was going through the list of treatment options, it didn't specify what the risks were of each. So, when I first went on Vancomycin and the Vancomycin taper like three or four times. I wasn't told ahead of time that hey, the more times you take Vancomycin, the greater the risk of your C diff coming back even worse.”*   - *Participant 2*   “*My doctor didn't have that information either, and I'm the one that brought it up. I'm the one that researched Dr. Kao and then when I took it to her, she says, yeah, OK and. So the lack of information out there in regards to the fecal transplant is, I find very alarming.”*   - *Participant 3* |
| 1. Thinking about your experience with rCDI/FMT, what information do you think is crucial for patients to know about rCDI/FMT? | What are the specific aspects about rCDI that should be emphasized in patient education materials? | Participants felt that the following are crucial information to know about rCDI:   - Causes of rCDI (including types of antibiotics that cause rCDI). - Signs and symptoms of rCDI. - There is a high possibility that CDI can reoccur. - The different treatment options, including FMT, and their risks. - When to seek treatment. - Having rCDI can affect your mental health. | *“I want to put there the types of antibiotics that might cause C diff, that triggers it.”*   - *Participant 5*   *“I think it would be important to have the different treatment options for like I mentioned this earlier, but having the different treatment options for when C diff not only occurs the first time, but the recur, when it occurs the multiple times after that and the risk of each treatment option would be important to have.”*   - *Participant 2*   *“I think the signs and the symptoms of it, if people know what to look for because I didn't know, I thought I'd eaten something bad and it wasn't that at all.”*   - *Participant 1*   *“If it was known that this is a high possibility of it reoccurring. That's good information to have and to be, I know I had to be an advocate for my own health because, I knew it, I wasn't better.”*   - *Participant 3*   *“For me it was not only challenging physically to deal with so much of it,* *but mentally as well. And there were times, I was thinking, oh my goodness. When is this going to end? So, it was affecting my mental health as well and I thought, every now and then, you know what I need to give up on this, this is just too much. So, for me, I feel that having information about not giving up, surrounding yourself with people you love, and who love you back, is important as well.”*   - *Participant 2*   *“I'm telling people…watch out for your type of antibiotic, it might flush all of your good and bad bacteria in your tummy and when that happens, and you have frequent diarrhea coming back. Go to your gastroenterologist and check out FMT.”*   - *Participant 5* |
|  | What are the specific aspects about FMT that should be emphasized in patient education materials? | Participants felt that the following are crucial information to know about FMT:   - There is hope and FMT works. - FMT isn’t as unpleasant as you may anticipate. - The preparation method for FMT is very similar to that of a colonoscopy. | *“It's important to just tell people. Yeah, there's hope this works.”*   - *Participant 1*   *“That it's nothing to worry about, actually cause it wasn't as bad as I thought it would be.”*   - *Participant 2*   *“I was very surprised at the preparation method for the FMT, [it] was very similar, if not identical, to a colonoscopy, and that to be perfectly honest, scared me because I haven't had a colonoscopy before. So that was a little nerve wracking to learn about that. But you know, having that information, there was I'm very happy I was provided with that information ahead of time, but having that info is important.”*   - *Participant 2* |
| 1. Are there any gaps or barriers in your rCDI or FMT care that have not yet been discussed? |  | Participants reported the following gaps or barriers in rCDI or FMT care:   - Lack of awareness of FMT by physicians. - Delays in having FMT presented as a treatment option. - Patients quickly received Vancomycin but were not told of its potential risks. | *“I have PBC and that's the start of why I ended up with C diff. But it's awareness and people don't know what PBC is, just like they don't know where its what C diff is and. I don't know where you start, I somehow think it's at the doctor level, if doctors knew more about it.*   - *Participant 3*   *“It took a while. A lot of Vancomycin, Metronidazole, testing, before I got referred to the FMT or to the clinic.”*   - *Participant 5*   *“It took them a very quick* [time] *to make a very quick decision to give him a Vancomycin, what it's called. It was a very quick decision on their part without letting us know that there are, there might, there will be a consequence if he stays on that medication, but that was a quick decision on that part. But sending him to FMT was a little bit slow.”*   - *Participant 4* |
| 1. In terms of accessing these patient education resources, do you prefer electronic formats (e.g. MyHealthAlberta) or hardcopy formats (e.g. brochures)? What are the reasons behind your preference? |  | Participants had mixed preferences for accessing the patient education resources.  Electronic resources:   - AHS website - Ads on social media that refer people to [Alberta Health Services](https://www.albertahealthservices.ca/) or [My Health Alberta](https://myhealth.alberta.ca/) - Short ads on TV - Animated YouTube video   Participants also mentioned hard copy resources for those who may not be tech savvy:   - Pamphlets with a catchy title and information on FMT - Pamphlets should be placed in gastroenterologists’ offices, pharmacies, and other physicians’ offices. | *“For digital resources, I feel that since a lot of people are using social media these days, like TikTok, Instagram, Facebook, all the other sites having. I think it was* [Participant 4] *who mentioned having little ads on there on TV, I feel that having little ads on social media as well that refer the user to go to the Alberta Health Services website or My Health. That can help as well.”*   - *Participant 2*   *“I love those small pamphlets that you take from a plastic thing, and then you open it three folds and then information’s over there on FMT and then the marketing would be on the front page would be something like ‘been pooping lately?’. And then at the bottom something like ‘try FMT’ and then as you open the page ‘what is FMT?’, with some short information on it. Because I got lots of that before* [my] *kidney transplant. And boy, did I learn a lot from it.”*   - *Participant 5*   *“At pharmacies, for example, since a lot of people do go to pharmacies to fill out their prescriptions, get blood tests, vaccinations, et cetera, but also in doctor's offices. Would be great.”*   - *Participant 2* |
| 1. Is there any other feedback or suggestions you would like to provide regarding patient education resources for rCDI and FMT? |  | Other feedback provided with regards to the patient education resources:   - Participants reported that there was a lack of information, despite the prevalence of this issue. - Create material for healthcare professionals at all levels to help increase their awareness and knowledge regarding rCDI treatment. | *“Once you have this and you start talking to different people, a lot of people have had C diff. Mine was a stubborn one. It took forever to get rid of, but there's a lot of people like that out there also. And just I think the lack of information about it was what I ran into.”*   - *Participant 3*   *“I think all the way around is education for everybody, like all levels, people have got to know about this.”*   - *Participant 3*   *“Have something there or have something sent out to the doctors. Like bulletins. Did you know? Or this. So, in case they don't know what to look for.”*   - *Participant 1* |

**Supplementary Table 2.** Video Script Initial Draft.

| **Script** | **Storyboard** |
| --- | --- |
| **What is *C diff?*** *Clostridioides difficile*, also known as *C diff, is* a bacterium that causes swelling or inflammation of the large intestine. Common symptoms include diarrhea, abdominal pain, nausea, loss of appetite, and even weight loss. | Visual of the anatomy of GI tract, show the bacteria colonizing the intestine |
| **You may be wondering, what causes this infection?**  Normally, there are trillions of good bacteria living in our large intestine. They help us digest food, and also keep out the bad bacteria.  When there is no longer a balance of the good bacteria in our gut, C diff can grow and take over. Antibiotics can usually cause this imbalance because they can kill the good bacteria! | Stay on the “Other risk factors” animation page and transition into the image of the good bacteria in our gut. |
| This imbalance is called “Dysbiosis”. Other things that may put you at risk of C diff infection include previous infection with *C diff,* being 65 or older, having a weakened immune system, or recently visiting a hospital! | Images of the ‘*C diff’* aka bad bacteria will show up amidst the ‘good bacteria’ while this is being narrated.  The word “dysbiosis” will show up on top of the previous image. |
| *No voice* | Transition slide |
| **So; how do you treat C *diff?***  Ironically, the way we treat C diff infection is by using another antibiotic, such as vancomycin. This treatment will kill some C diff bacteria, but it will also kill good bacteria at the same time. | A picture of antibiotics will come up, and transition into the next slide |
| For most patients C diff infection will resolve with a course of antibiotics. However, for some patients, the infection can come back again and again. It is now called ‘Recurrent *C diff. Infection”. The timeline is usually 2-4 weeks after you have finished vancomycin.* | The words: ‘recurrent *C diff’ pop up on the screen* |
| *No voice* | The text transitions from ‘recurrent *C diff’* to ‘Other Risk Factors’ and animated images of antibiotics, a patient in a hospital and an elderly population will show up. |
| **So if antibiotics can’t get rid of the infection, what do you do?** This is where Fecal Microbiota Transplant, or FMT comes in. FMT allows us to transplant the good bacteria from a healthy donor, into your gut. | New bacteria (supposed to illustrate donor bacteria) will show up on the slide, and subsequently the bad bacteria aka the *C diff* will leave. |
| FMT is regulated by Health Canada for the treatment of recurrent *C diff infection* to ensure everyone involved is safe and protected from harm. | The Health Canada logo pops up with an animation of EMR being screened. |
| **A common question is about *how* we ensure FMT occurs in a safe environment for the benefit of both the donor and patient.**  Stool donors are very carefully screened to make sure that their stool does not cause an infection in a recipient.  Donors are excluded if they have conditions like Irritable Bowel Syndrome, Inflammatory Bowel Disease, and Family History of colon cancer, just to name a few. | Three circles with “Irritable Bowel Syndrome”, “Inflammatory Bowel Disease” and “Family History will show up. They will subsequently be crossed out with an X on top of them. |
| Screening also includes tests for any harmful bacteria and viruses, such as E coli, Salmonella, HIV, hepatitis B or C, COVID-19 or monkeypox in the donor, among many others. | Three animated images show up of bacteria, COVID-19 and an individual with monkeypox. |
| FMT can be given by a colonoscopy (tube through your rectum), Gastroscopy (tube through your mouth), capsules (you can take them like medicine), or a feeding tube (when a machine sends food from a bag into your body through a tube). | 4 animated images of colonoscopy, gastroscopy, capsules and feeding tube come up, with corresponding text for each aligned with the method of administration being spoken about. |
| What should you expect after FMT? | Text of “What should you expect” pops up. |
| The success rate after FMT is very high, at least 80% after a single treatment. Some patients may need a second FMT.  Some common side effects include: abdominal pain, bloating, constipation and nausea. These symptoms are usually short lived and go away on their own. | Animated image of a woman with abdominal pain, stomach with pain animation to signal constipation and a woman throwing up are shown to accompany the text. |
| How can you tell the FMT has worked? | Text of “How do you know if the FMT was successful” pops up. |
| If your diarrhea does not come back, you can call yourself healed! | The diarrhea animated image used in the previous slide pops up with a red ‘X’ through it. |
| While you are getting better, it is important to take care of yourself and those around you. To stop the bacteria from spreading, make sure you are cleaning the bathroom often, and with bleach!  If you can use a separate bathroom, that is even better! | Animation of bacteria trying to spread, but the bleach and cleaning materials stopped it from spreading.  *Transition*  Show two bathrooms with one having a sign that says “*C diff only!”* |
| What can you do to make sure *C diff* does not come back again? | Text shows up that says “What can you do to prevent *C diff?”* |
| Avoid unnecessary antibiotics. If you do need to take antibiotics, only take the lowest dose you need to feel better! Always listen to your doctor and pharmacist, and avoid mixing drugs, and remind your doctor of your C diff history. | Three animated images show up:   - Antibiotics - Medicine bottle with Rx (to illustrate dosage) - Image with two kinds of drugs. |
| Lastly, make sure you're eating healthy! That’s the best thing you can do for your gut. When you eat fiber, fruits and veggies, you are also feeding your gut bacteria! | Animation with animated images of fruits, veggies and a fiber showing up. Sugar and fatty foods will show up after, but later disappear leaving only fiber, fruits and veggies in the frame. |
| *No voice* | Frames with logos of stakeholders and institutions needed to give credit to. |

**Supplementary Table 3.** Focus Group 2 Responses.

| **Question/ Category** | **Sub-questions** | **Summary of responses** | **Sample quotes** | **Suggested edits** |
| --- | --- | --- | --- | --- |
|  |  |  |  |  |
| **General Impressions** | After watching the video, what are your overall impressions? | - Participants found the video to be clear, to the point, and answered questions that they would have as a patient. - Participants thought the video was easy to listen to, user friendly, with no medical terminology that was difficult. - Participants thought that having the narration done by the male and female speakers gave the video variety and interest. However, they indicated that there was a difference in the volume of the male and female voices. - Participants also mentioned that the video moved fast in some areas. | *“I found it to be very clear and to the point, effectively conveying the most important information. So, it basically answered most of the questions I would have if I were still a patient and I appreciated that it explained the screening process for donors since this can provide additional comfort to patients.” - Participant 2*  *“I think that it's very user friendly, even for a variety of ages, right? It's not jargon. It's not medical jargon. It's not too simple. It's not too complex. I really liked it and I thought the images were really useful as well.”*   - *Participant 6*   *“It was nice though, to have the two speakers, like it sounded collaborative. I like that it was narrated by two different people. It gave it a little bit more variety and interest I'd say.”*   - *Participant 6*   *“The audio of the girl is much higher volume than the guy...”*   - *Participant 5*   *Like [Participant 6] mentioned, it's like it went fast that the picture went and it's like ohh no, how it's one of those again. It’s too much, I can't follow.”*   - *Participant 5* | Use the exact same method to record the audio for both narrators to make sure that their audio quality and volume are the same. |
|  | Did it capture your attention and keep you engaged? | All three participants said that the video captured their attention and kept them engaged. | *“Ohh yes it did, yeah.”*   - *Participant 2* | N/A |
| **Clarity** | Was the information presented in the video clear and easy to understand? | Participants indicated that the information presented in the video was clear and easy to understand. | *“Yes, I would say it was easy as I follow through and understand the language, it's like I'm going through the process again and every word that was mentioned, I go, ‘Yes, that happened.’ ‘Yes, that's what it is.’ So, I felt the whole video carried me through my whole journey.”*   - *Participant 5* | N/A |
|  | Were any parts of the video confusing or unclear? | While participants said that there were no parts of the video that were confusing, they recommended adding a timeframe to the portion of the video that talked about recurrence of CDI to increase clarity.  Participants indicated that the video made it seem like cleaning the bathroom only needs to occur after treatment with FMT. | *“For myself, I found the information to be quite clear. However, there was one specific question. ‘How do you know if the FMT was successful?’ and the answer was if the diarrhea does not come back, then you can consider yourself cured…But I found that to be quite vague, since there is no window of recurrence specified.* *So, if the diarrhea does not come back in three days are they cured? or three weeks? etcetera.”*   - *Participant 5*   *“The part where, after the transplant, she said ’while you're getting better, make sure that you clean your bathroom, and you use a separate bathroom.’ But that's also applicable before [keeping your bathroom clean]…that should kind of be a general C diff statement, not just after transplant.”*   - *Participant 6* | Add a timeframe to the video that gives more information about when to consider CDI as recurrent.  Clarify that cleaning the bathroom occurs all throughout having CDI. |
| **Plain Language** | Did the video use language that was easy to understand? | Participants reported that the video used language that was easy to understand. | *“Yeah, for me there were no occurrences where I was thinking, ‘what do they mean when they said that?’ I understood everything completely.”*   - *Participant 2* | N/A |
|  | Were any medical terms or concepts difficult to grasp? | All participants agreed that there were no medical terms or concepts that were difficult to grasp in the video. | *“It's not jargon. It's not medical jargon. It's not too simple. It's not too complex.”*   - *Participant 6* | N/A |
| **Amount of Information** | In your opinion, did the video provide the right amount of information about Clostridium difficile infection? About fecal microbiota transplantation? | - Participants felt that the amount of information presented in the video about CDI and FMT was appropriate for the length of the video. - One participant thought the information in the video was purposeful and intentional. - Participants thought the video could be longer. | *“Yeah, but the content for the time was good.”*   - *Participant 5*   *“It didn't drag in any sections; it was very purposeful and intentional about what was being spoken.”*   - *Participant 6*   *“…for information with this much value, I don't think it would be much of an issue to extend the video by maybe another minute or so.”*   - *Participant 2*   *“…you can add more. I'm OK with what P2 said, add one more minute. I did feel like at the end of the presentation, like that's it? Kind of short. So yes, I would like to hear some more.”*   - *Participant 5* | Add more information about CDI and FMT – see suggested edits below. |
|  | Was there anything you felt was missing or needed more detail? | - Participants agreed that the risks of FMT were not covered, and it would be important to add this information. - One participant pointed out that the lists of symptoms for CDI and after treatment with FMT are similar and to clarify by specifying symptoms after FMT and listing the symptoms. - There was no explanation of how the fecal matter is processed after it is received from the donor. | *“I'm not sure if I saw this in the video, but what are the risks of the FMT…? I don't think that was covered.”*   - *Participant 2*   *“…the phrase in the video said ‘some symptoms’ and then they said bloating, gas, constipation. This was that list, but that list [common symptoms of FMT] actually has some similarities, I mean apart from the constipation, to the very first list about just C diff in general, and they didn't specifically say ‘some symptoms after [FMT] treatment’., I think it would help to clarify to say ‘some symptoms after FMT’ or ‘after treatment’ and then list those symptoms – [this] would help to clarify that a little bit from my perspective.”*   - *Participant 6*   *“…we could potentially think about adding a little bit…about…how it goes from donor to the capsule or to, whatever form you're using. So, it might be valuable to say that this is fecal filtrates, or that it's processed in a certain way…or whatever you want to say. We don't have to get scientific, but it would be nice so that they know there's a process to refining it so that it's not just stool to you, right?”*   - *Participant 6* | Add the risks of FMT.  Add “of CDI” and “of FMT” to the phrase “common symptoms.”  Add a high-level explanation of how the fecal matter is processed after it’s received from the donor and eventually delivered to the patient. |
| **Pace of Information** | What did you think about the pace of the video? Was it too quick, too slow, or just right? | All participants reported that there were portions of the video where the audio moved quicker than the images and where the slide transitions were very quick. They also felt like the pace of the video can be slowed down especially for people who have a hearing impairment or are less comfortable with technology. | *“Yeah, I think it was mentioned by both P5 and P6 earlier. But yeah, I found the, like especially I think it was in the first minute to 1st 90 seconds of the video, the slide transitions were very quick and someone mentioned that it was, like in, there were examples where they were looking at the visual element by the time they were able to see all the parts of that element, it was already gone to the next slide.”*   - *Participant 2*   *“I thought it was. It was a good pace for the most part, but there were a few elements that moved too quickly for my preference. But also, I'm thinking if you had someone that maybe wasn't as tech savvy or is hard of hearing or has to adjust things like, umm, just even that processing of listening it could be pretty quick depending on the demographic of who's listening.”*   - *Participant 6* | Ensure images are synced with the audio so that they move together.  Slow down slide transitions.  Slow down the pace of the video a little. |
| **Visual and Audio Elements** | In your opinion, were the visuals effective in helping you understand CDI? FMT? | All the participants agreed that the visuals were effective in helping them understand CDI and FMT. | *“They were [effective]. Well, for me they were…”*   - *Participant 2* | N/A |
|  | Was the audio (voice over) effective in helping you understand CDI? FMT? | - Participants reported that the audio was effective in helping them understand CDI and FMT. However, they all indicated that the male and female audio were not the same quality and volume, and therefore, not balanced. - Participants indicated that they enjoyed the narrative style of the video. - Participants noticed that while the video was animated, there were photos that were viewed as inconsistent with the rest of the video. They also mentioned that the image was of fried food, and this is not the best representation of the type of meals that a CDI patient should be eating. One participant mentioned that the food shown in that image also has cultural connotations and may not resonate with some people. | *“Yeah, I noted that same thing as well, where the female narrator was louder than the male narrator. I was constantly having to fiddle with the volume controls on my laptop just to compensate for that.”*   - *Participant 2*   *“I thought the narration was really great and I liked that it was speaking more than just the words on the screen because it added to it...I liked it.”*   - *Participant 6*   *“…the whole video was like cartoony and that wasn't [the photo of the food]. And that seemed odd also.”*   - *Participant 6*   *“…there was one element at the 29 second mark that shows what appears to be a lot of fried foods or some sort of buffet which people should not be eating anyways. So, to improve the optics I recommend replacing that.”*   - *Participant 2*   *“And there was also something about that particular image that almost had, like a cultural implication to me, just with the foods type. And I think that could be limiting to some people feeling like it resonates with them.”*   - *Participant 6* | Use the exact same method for audio for both speakers to make sure that the audio is consistent and balanced.  Keep video consistent – that is, if animated, use animated images throughout. Remove still photos and replace with an appropriate animated image.  Replace visual of fried food with foods which CDI patients should be eating including raw fruits, vegetables, and high fiber foods.  Keep in mind that some foods have cultural implications and may not resonate well with some people. |
|  | What aspect of the video did you like the most or find the most useful? Why? | Participants reported that they enjoyed every aspect, from the animated style to the narration, images, and how the video progressed. They also agreed that presenting the multiple ways that people can receive FMT was good. | *“I really enjoyed the style of it, the narration, the images, and the flow. I thought it was really exceptional and [will] be a really valuable tool.”*   - *Participant 6*   *“I really like the progression from describing what C diff is to how the donors are selected in the screening, and then after… how it's administered. I thought the progression was really great.”*   - *Participant 6*   *“When I think about it, I felt that it was nice that the delivery mechanism was there because there are the people that are not entirely comfortable with swallowing capsules, for example, or, you know, having to undergo a colonoscopy. So, I felt it was great that there were the multiple delivery options that were presented.”*   - *Participant 2* | N/A |
|  | What aspect of the video did you find the least useful? Why? | - Two participants agreed that the QR code may be the least useful part of the video. They felt like it may be too ‘techy’ for some audiences. - One participant liked the QR code but suggested that to cater to various demographics, more options for information should be presented. | *“Well, if I can be honest, maybe the last part, the QR code…Something like that, because I'm not the techy. I don't know how to do that QR code...”*   - *Participant 5*   *“I personally really like the QR codes. I think that it's really valuable to have a source to go to for more information, but I really like your point that I think you have to cater to the different demographics that might be viewing this. So, you have to have a phone number. You have to have a website. You have to say, you know, speak to your primary care provider, and I think a QR code is valuable for those that know how to use it. But, I guess, to not accentuate that any one of these modes is more valuable than the next. It’s just here - for more information, here are your places to go.”*   - *Participant 6* | Present more options to find information in addition to the QR code (e.g. webpage links to MyHealth Alberta, Fecal Microbial Transplantation Clinic phone number). |
| **Diversity and Inclusion** | In your opinion, did the video consider diversity and inclusion in its content and presentation? For instance, did it represent the different types of people that may be affected by CDI? | - All participants agreed that the video was diverse and inclusive in its content and presentation. They felt like everyone who can be affected by CDI were represented in the video. - One participant did not like the word “patient” on the slide with the washrooms as it felt like it has connotations to being hospitalized. They also felt like having signage showing male and female restrooms was not adding anything to the slide. | *“Yeah, to me it seemed like the video portrayed people of different ages, skin colors, gender identities, and even the narrators were male and female. So yeah, I think it was, I have no concerns about representation and diversity and inclusion.”*   - *Participant 2*   *“Exactly because…the bathroom signs that were there for two different doors. And then the one sticker that went on was like C diff patients only, and it, there was something about that that just felt like hospital-ish or like institution just being labeled patients.”*   - *Participant 6*   *“Having both of those doors have male and female symbols. I don't think that adds anything if you just had, like a toilet sign or whatever, right? Or washroom. Or even if you don't want to write washroom, that it's something that someone has to read. But just having a toilet on there, it changes it.”*   - *Participant 6* | Remove “patient” from the slide with the washrooms and just have signage that indicates a separate restroom.  Replace the signage that shows male or female with a toilet icon. |
|  | Do you think the information in the video will be understood by people of various backgrounds? | All participants agreed that the information in the video will be understood by people with various backgrounds. However, two participants were not sure if they would have understood the information in the video if they had not already experienced CDI. | *“…I would say [I learn more slowly] and I really enjoyed it. I loved it.”*   - *Participant 5*   *“…yeah, for me it was quite easy to understand.”*   - *Participant 2*   *“I think that the one that's not gonna understand that is the one that didn't undergo C diff. Because like I said, it carried me through. My whole journey was, it's in that content, so, I understand. My mind really enjoyed it.”*   - *Participant 5* | Add high-level content at the beginning of the video to make it easier for people who may not have gone through the CDI experience. Share the broader implications of having CDI, its impact on different aspects of people’s lives, how it can be all-consuming and affect the lives of those around the affected person (for example, family members). Suggested content areas to add include emotional aspects, common fears, misconceptions, concerns of spreading CDI to loved ones, etc. |
| **Emotional Impact and Confidence** | If you had not already gone through FMT, would you feel more informed about understanding FMT as a treatment option for CDI? Would you feel more confident? | All participants agreed that the video would have been a valuable resource for them if they had not gone through FMT already. | *“I wish this video was around when I was actually dealing with C diff. I mean it would have saved me a lot of time googling and scaring myself. I feel this would have been valuable.”*   - *Participant 2* | N/A |
| **Feedback on MyHealth Alberta Format** | Participants were shown the following examples of health information on MyHealth.Alberta.ca:  [Early Hearing Detection - Screening in Infants (alberta.ca)](https://myhealth.alberta.ca/Alberta/Pages/early-hearing-detection-screening-infants.aspx)  [Colonoscopy video series (alberta.ca)](https://myhealth.alberta.ca/colonoscopy)  [FIT_Step_by_Step_Instructions.pdf (screeningforlife.ca)](https://screeningforlife.ca/wp-content/uploads/FIT_Step_by_Step_Instructions.pdf)  They were asked for feedback on what was most helpful and least helpful on the webpages shown. | - Participants liked the Q&A style for presenting information. - The majority of the participants preferred video format. Additionally, one participant did not want a lot of reading. - The majority of the participants preferred having the health information presented in multiple formats to cater to multiple audiences and contexts – for example, people who may not be able to watch the video in a public space can read a transcript of the same information in the video. | *“I really like the Q&A style that's then augmented by having the video, which is further augmented by having that transcript, if that's how you prefer to learn, or hear it, or view it, or whatever.”*   - *Participant 6*   *“…and then when I saw #3 [the FIT infographic], I said, whoa, that is awesome diagram, but then I see a lot of things to read. But then I…go back again to #2 [the Colonoscopy video series] because I will play the video and I will see pictures and I will hear the teacher explaining…I like instruction, actual video, and instructions...The least one I like is #1 [Early Hearing Detection] cause there's a lot of reading and there's a link that I can click on, but then it leads to another reading, and I think that's where I will pass it to my wife and say, sweetie, can you explain this or read this for me? So, I'm gonna go back to #2 [the Colonoscopy video series] as my favorite one. [I] watched the video, and I just learned a lot from watching it.”*   - *Participant 5* | Present the information as Q&A style in multiple formats – a readable transcript with videos embedded prominently (or link to a YouTube video).  Link to an infographic or other handout. |
| **Other Feedback** | Participants were asked to give any other feedback or suggestions about the video. | N/A | *“…they didn't really touch on what recurring [means]…one slide just says if antibiotics can't get rid of the infection, what do you do?...They didn't really go into what does that mean if it doesn't get rid of it? Like what is ‘recurring’ because recurring really is the key to being eligible for FMT.”*   - *Participant 6*   *“I didn't love that they said ‘vansomycin’.”*   - *Participant 6*   *“…at 18 second mark and it just says symptoms include diarrhea, abdominal pain, nausea, slash, loss of appetite. But really that could be two separate ones…”*   - *Participant 6*   *“It might be valuable to add more logos either at the beginning or at the end. I think to just legitimize the source a little bit. So, it’s either like Alberta Health or Alberta Health Services or University of Alberta, like something that's more obvious to know who made this video and that I think it instills a little bit more confidence in the legitimacy of the information.”*   - *Participant 6*   *“…it would be nice if it ended with the QR code so that they stayed there…I had to go back and pause it to be able to get my phone out to do those QR codes.”*   - *Participant 6*   *“But he says if you have more questions, this is a QR code, and I didn't really like that statement…maybe change it to ‘for more information’ go here. It's different to me because some might not have questions, but they might just be curious to read more from another source.”*   - *Participant 6*   *“I think there is a bit of an opportunity…about maybe expanding on the pros and cons of the different FMT options, but that's probably getting into a whole other thing...”*   - *Participant 6* | Define ‘recurrence’.  Change pronunciation of Vancomycin.  Separate ‘nausea’ and ‘loss of appetite’ as separate symptoms.  Add logos to legitimize the source of the information.  End the video with where to find more information and keep the QR code on the screen.  Change the wording of “if you have more questions” to “for more information”.  Remove the Canva watermark on the final version of the video.  Consider the potential of a video series similar to the Colonoscopy video series. Example videos can focus on the pros and cons of the ways that FMT is administered, give more details on the different aspects of having recurrent CDI, etcetera. |

**Supplementary Table 4.** Video Script Final Script.

| **Script** | **Storyboard** |
| --- | --- |
| In this video, you’ll learn about the process of Fecal Microbiota Transplantation (FMT) for treating recurrent C. *diff* infection. You’ll see what C. *diff* is, what causes it, and how it's typically treated. Then, you’ll learn how FMT products are prepared and made safe for patients. Finally, you’ll learn the benefits and risks of FMT along with important strategies for preventing C. *diff* infection.  **What is *C diff?*** *Clostridioides difficile*, previously called Clostridium difficile and also known as *C diff, is* a bacterium that causes swelling or inflammation of the large intestine. Common symptoms of a C diff infection are diarrhea, abdominal pain, nausea, loss of appetite, and weight loss.  C *diff* can make you very weak and can also lead to social isolation, fear of spreading it to others, or stress. | Visual of the anatomy of GI tract, show the bacteria colonizing the intestine   - Show swelling/ inflammation so people can visualize this internally. - Have ‘C diff’ show up on the screen so the spelling can be visualized. |
| **You may be wondering, what causes this infection?**  Normally, there are trillions of good bacteria living in our large intestine. They help us digest food, and also keep out the bad bacteria.  When the good bacteria in our gut are no longer balanced, C. diff can grow and take over.  So what causes this imbalance? Taking antibiotics is the most common cause, because they can also kill our good bacteria. This imbalance is called dysbiosis. | Visual of “good” (green bacteria) and “bad” (Red bacteria) |
| Other things that may put you at risk of C. diff infection include: previous infection with *C. diff,* being 65 or older, having a weakened immune system, or having recently had a hospital stay. | The text transitions from ‘recurrent *C diff’* to ‘Other Risk Factors’ and animated images of antibiotics, a patient in a hospital and an elderly population will show up. |
| *No voice* | Transition slide |
| **So; how do you treat a C *diff* infection*?***  Ironically, the way we treat C diff infection is by using another antibiotic, such as vancomycin. These antibiotics will kill some C diff bacteria, but they will also kill good bacteria at the same time. Some C diff bacteria are also hard to kill with antibiotics because they can go dormant by forming spores to evade antibiotics. | A picture of antibiotics will come up; some C diff bacteria get killed but others form spores and cannot be killed by antibiotics. |
| For most people, C. diff infection will go away with a course of antibiotics. But for some people, the infection can recur, meaning it can come back again and again. This is called “recurrent C. diff infection”. This means that while you take vancomycin, your diarrhea clears up, but after you stop vancomycin, C diff and diarrhea come back. If the infection comes back, it usually comes back 2-4 weeks after you have finished vancomycin. | The words: ‘recurrent *C diff* infection*’ pop up on the screen.* Vicious cycle of C diff and diarrhea, going on vancomycin with diarrhea resolution, but comes back after stopping vancomycin. |
| **How do you treat a recurrent C. diff infection?** If you have a recurrent C. diff infection, vancomycin can be like a bandage: it may stop some symptoms but it is not treating the actual problem. To get past the band aid effect of vancomycin, we use Fecal Microbiota Transplantation, or FMT. FMT allows us to transfer good bacteria from a healthy donor into your gut. | New bacteria (supposed to illustrate donor bacteria) will show up on the slide, and subsequently the bad bacteria aka the *C diff* will leave. |
| FMT is regulated by Health Canada for the treatment of recurrent *C diff infection* to make sure both the donor and the recipients are safe and protected from harm. | Animation of health screening tools. |
| **A common question is about *how* we make sure FMT is safe.**  First, stool donors are screened very carefully to make sure that their stool will not cause an infection in a recipient.  Donors are not able to donate stool if they have conditions like irritable bowel syndrome, inflammatory bowel disease, or a family history of colon cancer, just to name a few. | Three circles with “Irritable Bowel Syndrome”, “Inflammatory Bowel Disease” and “Family history of colon cancer” will show up. They will subsequently be crossed out with an X on top of them. |
| Second, the donor stool is tested for any harmful bacteria and viruses, such as E. coli, Salmonella, HIV, hepatitis B, hepatitis C, COVID-19 and mpox, among many others.  It may be reassuring to know that there are actually more tests done on donor stool than are done on donated blood! | Three animated images show up of bacteria, COVID-19 and an individual with monkeypox. |
| **How is the FMT treatment made?**  The stool donation from a screened donor is mixed with water and then the large undigested particles are filtered out. This creates a liquid suspension with good bacteria. This suspension is freeze-dried so it becomes a powder which can then be packaged into capsules or re-suspended in water, depending on how the treatment is to be given.  **How is FMT given?**  The good bacteria from a donor can be transferred to your gut by a colonoscopy (a tube through your rectum), gastroscopy (a tube through your mouth), capsules (medicine that is taken by mouth), or a feeding tube (a machine sends food from a bag into your body through a tube). There are benefits and drawbacks to each delivery method. For example, taking FMT by pills is the least invasive, but this will not work if you have trouble swallowing pills. Giving FMT by colonoscopy is more invasive, but it may also allow your healthcare team to find other problems such as inflammatory bowel disease or bowel cancer that they would not be able to detect otherwise.  **What are the risks of FMT?**  There is a risk of getting an infection from the donor stool despite all the testing, especially if there is an infection that we currently do not have a test for. Because it is a relatively new procedure, the long-term consequences of FMT are not yet fully known.  We always have to consider the risks and the benefits of any medical treatment. For FMT, the benefits far outweigh the risks. | Animated images of FMT production, from stool, to liquid slurry, to a lyophilizer, then into powder which can be made into capsules or resuspended in water.  4 animated images of colonoscopy, gastroscopy, capsules and feeding tube come up, with corresponding text for each aligned with the method of administration being spoken about.  Animated images of potential spread of an infection and unknown risk of FMT.  Show an animated image of a small circle of “risk” and a large circle of “benefits” to emphasize the fact that the risks are very small. |
| **What should you expect after FMT?** | Text of “What should you expect” pops up. |
| The success rate after FMT is very high. At least 8 out of 10 people don’t have another C diff infection after a single FMT treatment. Some people may need a second FMT treatment.  If your diarrhea does not come back 8 weeks after your FMT, you can consider your recurrent C. diff infection cured!  Some common side effects of FMT include abdominal pain, bloating, gas, constipation, and nausea. These symptoms usually don’t last long and go away on their own. | Have a visual of 8/10 patients being extremely happy and satisfied with their health following treatment.  ****Focus on the success rate, not the side effects.*** |
| **What are ways you can help to stop the spread?** | Text shows up that says “What can you do to prevent *C diff?”* |
| To stop the bad bacteria from spreading, clean your hands often with soap and water. Also, make sure you clean the bathroom regularly with bleach while you still have C. diff diarrhea.  If you can use a separate bathroom while you have a C diff infection, that is even better!  To make sure your C. *diff* infection does not come back, avoid unnecessary antibiotics. For example, a cold is most often caused by a virus and does not need antibiotics. If you do need to take antibiotics, remind your doctor of your C. diff history, and request a low dose, narrow spectrum antibiotic if possible! Always listen to your doctor and pharmacist, and avoid mixing medicines. | Animation of bacteria trying to spread, but the bleach and cleaning materials stopped it from spreading.  Show 2 bathrooms with one having a sign that says “C diff only!”  Animation of a patient talking to a doctor/ pharmacist. |
| Lastly, make sure you're focused on eating nutritious meals! That’s the best thing you can do for your gut. Eating a balanced diet with plenty of fiber, including fruits and vegetables, allows your good gut bacteria to keep growing! | Animation with animated images of fruits, veggies and fiber showing up. |
| Thank you for watching this video! For further information, visit [website] to learn more about FMT, or talk to your local healthcare provider! | Frames with logos of stakeholders and institutions needed to give credit to.  Add disclaimer re: medical information |

**Supplementary Table 5.** Focus Group 3 Responses.

| **Question/ Category** | **Sub-questions** | **Summary of responses** | **Sample quotes** | **Suggested edits** |
| --- | --- | --- | --- | --- |
|  |  |  |  |  |
| **Audio elements** | In the second focus group, all participants mentioned that there were some issues with the audio. After watching this new video, what are your thoughts about the audio quality? | Participants reported that they did not have any issues with the audio quality of the video. | *“Yeah, I agree. I think that was fully remediated in my opinion. Don't have any concerns related to that.”*   - *Participant 6* | N/A |
|  | What are your thoughts about the volume level of both speakers? | One participant indicated that there was a slight difference in the volume level of the male and female voices. | *“Audio is good. But in the beginning I watched it on my cell phone and I was up and down on my volume on the cell phone. Maybe if it's just a little bit more tad volume for the lady’s volume, but just a little very, very little to none. I would say that less. But when I watch it on the computer one, it's perfect.”*   - *Participant 5* | Increase the female’s volume level a little. |
| **Clarity** | Is the timeframe of what defines recurrent CDI clearly explained in the video? | - Participants indicated that the timeframe that defines recurrent CDI was clearly explained. - However, there were a couple of sections that participants felt needed improvement in the wording of some sentences. | *“Yup. That's one of the things I took note of is that they did mention the eight week recurrence window. So yeah, I don't have any more concerns about that.”*   - *Participant 2*   *“…specifically to that section, I would say my only comment is that I think that there's room to improve on that initial question. So I think the question said what can you do to stop the spread and ensure it doesn't come back again? Something like that. And I think that statement alone is a bit too prescriptive, like factual, because sometimes no matter what you do, it may come back. And I think that phrase makes it seem like you did something wrong if it comes back. it's almost like if you do this, it will not come back. So I think perhaps to change that phrase in some way to maybe perhaps say like what are ways you can help to stop the spread and make it not come back or whatever instead of saying what can you do to stop the spread?”*   - *Participant 6*   *“…at the 8:19 mark, there's a statement that says pick an antibiotic that only works with specific bacteria. And I feel that could be misleading, since the way it's worded it may imply that the viewer rather than the healthcare provider, is picking the antibiotic for the patient. So I would change that a little bit.”*   - *Participant 2* | The answer to what can you do to stop the spread and ensure it does not come back should be rewritten. As it is right now, it makes it seem as if CDI reoccurs, it’s the person’s fault. Suggestion: what are ways you can help stop the spread?  The statement that says “pick an antibiotic that only works with specific bacteria” may be misleading and should be rewritten to clarify that the provider is the one prescribing the antibiotic and not the patient. Also, clarify that the patient can request a low dose, narrow spectrum antibiotic. |
|  | Is the information regarding cleaning of bathrooms clear and easy to understand? | All participants agreed that the information regarding the cleaning of bathrooms was clear and easy to understand. | *“It was quite clear and it was to the point. there were no mincing of words in regards to it.”*   - *Participant 2* | N/A |
| **Amount of Information** | Did the video provide the right amount of information about the risks of FMT? | Participants agreed that the video provided the right information about the risks of FMT. However, one of the participants mentioned that the video made it seem like the risks of FMT are unknown but a lot of the risks of FMT are known | *“Yeah. I didn't expect anything more than that. I felt it covered it very well in terms of specifically the composition of the FMT capsules, which was interesting to see.”*   - *Participant 2*   *“One thing that jumped out to me was I think, he said risks are not yet known, even though he said there's some risks and we do a lot to screen, I would maybe suggest putting risks not fully known because we know that there that we know a lot. When we say not yet known, it sounds like we don't know very much, but we actually know a lot. So I would suggest saying risks not yet fully known.”*   - *Participant 6* | Change the sentence that says “risks are not yet known” to “risks not yet fully known.” |
|  | Did the video provide the right information about the processing of fecal matter for FMT? | The participants reported that the video generally provided the right information about the processing of fecal matter. They all agreed that the use of the term “fecal slurry” may be too much information. | *“And then that fecal slurry that was an interesting picture. Some people might find that too much information. I guess depending on the person, but in some ways the more you know it's better. So I don't have a strong opinion on that, but some people may struggle with like it's on the edge of like, that's too much, too much information.”*   - *Participant 6*   *“I would go with maybe just remove the slurry. The slurry paused me and made me think not slurry.”*   - *Participant 5* | Fecal slurry was seen as potentially too much information and could be toned down to eliminate the pictorial aspect.  Remove the word “slurry.” Suggested replacement is “suspension.” |
|  | Is the common symptoms list for CDI clear? Is the common symptoms list for FMT clear? | The participants indicated that the common symptoms list for both CDI and FMT are clear. | *“Yeah, I felt like that was improved. It was clear to me in this video.”*   - *Participant 6* | N/A |
| **Pace of Information** | What do you think about the pace of this video? Still too fast? Too slow?  Are there any portions where the audio moves quicker than the images? Are there any portions where the slide transitions are too quick? | All participants reported that the pace of information was mostly fine. However, there were still portions of the video where the audio was not aligned with the images. | *“I felt a pace of information was pretty good for most parts of the video. However, I did find that at very specific time stamps, the duration did not align with the visual elements. So specifically at 4:28, 6:50 and the 8 minute mark. So it was probably a difference of three to five seconds.”*   - *Participant 2*   *“I did see one. Yeah, well, it it's OK with me. But since I was looking at it, it's the word COVID. Mentioned and then the COVID picture. That was a little off, but I said, yeah, that's alright. So maybe two second delay. So I'm good with that 'cause it flashed anyway. Be careful of COVID, where’s COVID. Oh, there it is now. I'm good.”*   - *Participant 5* | Ensure images are synced with the audio so that they move together. |
| **Visual Elements** | What are your thoughts about the visuals? Are they appropriate for the audiences affected by CDI? | Participants agreed that the visuals were appropriate for audiences affected by CDI with the exception of the glass of fecal slurry. | *“I love the diagram especially that little yellow Pac-Man thing that's going [makes sound]. And the pictures are all very nice. I love it.”*   - *Participant 5*   *“Other than the glass or the jar of slurry, I'm OK with them.”*   - *Participant 2* | Remove the image of the jar of fecal slurry. |
|  | At the very end of the video, are the options presented appropriate for anyone who may want more information about FMT? Do you have any additions? | Participants agreed that the options provided were appropriate. One participant suggested that Health Link could be added as an option. | *“I would probably add health link’s number to that slide as well since some people may not, you know, be comfortable with e-mail or accessing a website and where most people in Alberta are promoted to contact health link for health information.* *So I think that would be a great touch point to promote health link, a little bit more by adding that number to the slide.”*   - *Participant 3* | Add Health Link to the resources slide. |
| **Diversity and Inclusion** | Is the signage for the restrooms appropriate? Are the icons appropriate? | All participants agreed that the signage and icons are appropriate. | *“I truthfully didn't notice anything about them.”- Participant 6* | N/A |
| **Emotional Impact and Confidence** | In your opinion, is there enough information presented that would help someone who has not experienced CDI understand the broad impact that CDI has on every area of one’s life? | All participants agreed that the video would have been a valuable resource for them if they had not gone through FMT already. | *“Yeah, like I said during the previous meeting, I wish this video had been available when I had C diff. So yeah, it was great.”- Participant 2*  *“It was good. It was all there, especially all those the added information like scared to transfer it to the caretaker, those four diagrams there, and then it carried through the whole story.”- Participant 5* | N/A |
| **Other Feedback** |  | N/A | *“Just that last slide. It didn’t represent well, kind of who this was coming through and who made this. I would just say the second to last slide. Like thank you, For more information visit, Contact us. …like it doesn't have a lot of what I would say, like professional logos, the very last one that is kind of like the disclaimer has a MyHealth.Alberta.ca and Alberta Health Services logo that looks way more legit, but that second to last slide looks too PowerPoint Ish. Not polished in my opinion.”*   - *Participant 6*   *“…when it was talking about the modes of delivery and saying capsules, like colonoscopy capsules. The phrase when it said capsules was taken like medicine. And I don't think that that's, like people take medicine in all different routes and so perhaps someone's a diabetic and their medicine comes in an injectable so it was just too broad to say, taken like medicine. So capsules may be taken by mouth.”*   - *Participant 6*   *“…one of the last slides was talking about fiber, fruits and veggies. It really only listed those three which I thought maybe a bit restrictive to maybe touch on those…focus on like a well-balanced or like nutritious [meals].”*   - *Participant 6*   *“…it was right around the six minute mark it's talking about who, who has C diff and there was a picture of someone in a hospital bed and I didn't love that the person who has C diff is pictured in a hospital bed because it kind of makes people think maybe like, Oh well, I'm not that bad. Or like it only happens in hospital or like that doesn't totally apply to me so maybe just throwing it out there to have it not such a image of inpatient treatment.* *It doesn't have to look like in a hospital bed, so I just don't want people to take that.”*   - *Participant 6*   *“Actually, the more I think about that, the more I like that even because also considering that vancomycin is extremely expensive. So if you have someone that has an option for, you know the physician saying we can do flagyl, we can do vancomycin and it's a cost factor. We don't want someone thinking that they're getting a subpar treatment because of a, you know, personal concern that they may choose one option or the other.”*   - *Participant 6* | Add the logos of all stakeholders to show who the partners are and to legitimize the source of the information.  Change the sentence that mentions “capsules taken like medicine” to “capsules taken by mouth.”  The slide that mentions fruit, and veggies should be rewritten to focus on well-balanced meals.  Change the image of the person with CDI in a hospital bed to one that shows a person with CDI in an outpatient scenario – example suggestion was the person could be sitting in a chair instead of laying in a hospital bed.  Participants wanted to know if Vancomycin is the standard of care since it was mentioned in the video but Flagyl was not. There may need to be an explanation in the video so that if patients receive a different antibiotic, they do not think they have received subpar treatment. |
